# 538. Clinical Evaluation of Drug-drug Interactions with Remdesivir

**DOI:** 10.1093/ofid/ofad500.607

**Published:** 2023-11-27

**Authors:** Chi-Chi Peng, Mark Shelton, Anuja Raut, Anna Kwan, Kanika Chhabra, Gong Shen, Deqing Xiao, Aryun Kim, Sharline Madera, Robert H Hyland, Helen Winter, Rita Humeniuk

**Affiliations:** Gilead Sciences, Inc., Foster City, California; Gilead Sciences, Inc., Foster City, California; Gilead Sciences, Inc., Foster City, California; Gilead Sciences, Inc., Foster City, California; Gilead Sciences, Inc., Foster City, California; Gilead Sciences, Inc., Foster City, California; Gilead Sciences, Inc., Foster City, California; Gilead Sciences, Inc., Foster City, California; Gilead Sciences, Inc., Foster City, California; Gilead Sciences, Inc., Foster City, California; Gilead Sciences, Inc., Foster City, California; Gilead Sciences, Inc., Foster City, California

## Abstract

**Background:**

Remdesivir (RDV), a nucleotide prodrug approved for COVID-19, is a substrate and inhibitor of organic anion-transporter polyprotein (OATP) transporters and cytochrome P450 3A4 (CYP3A4) metabolizing enzyme based on in vitro data. Here, clinical studies of potential drug-drug interactions (DDIs) assessed RDV as a victim of OATP1B1/B3 inhibition and CYP3A induction and RDV as a perpetrator on OATP and CYP3A4.

**Methods:**

Two Phase 1 open-label, fixed sequence studies were conducted in healthy participants (Table 1). Study 1 (n = 9) tested the effect of OATP1B1/1B3 inhibition (cyclosporin A, CsA) and CYP3A4 induction (carbamazepine, CBZ) on the pharmacokinetics (PK) of RDV. Study 2 (n = 19-22) tested the effect of RDV on the PK of substrates of OATP transporters (pitavastatin, PIT) and CYP3A4 (midazolam, MDZ). Plasma concentrations of RDV, its metabolites GS-704277 and GS-441524 (Study 1), and probe substrates (Study 2) were measured by validated mass spectrometry methods. PK parameters (AUC_inf_ and C_max_) were estimated by noncompartmental analysis (Phoenix WinNonlin™). Treatments were compared with a parametric mixed-effects model with point estimates for geometric least-squares mean (GLSM) and 90% CI with pre-specified no-effect bounds. Safety was monitored throughout.

**Results:**

In Study 1, coadministration of RDV with CsA resulted in 89%, 197%, and 3% increases in AUC_inf_ for RDV, GS-704277, and GS-441524, respectively, whereas coadministration with CBZ did not impact AUC_inf_ or C_max_ for RDV or its metabolites. In Study 2, RDV did not impact AUC_inf_ of PIT or MDZ, although mild increases in C_max_ were observed for MDZ and 1’-OH-MDZ (29% and 25-39%, respectively). No deaths, serious adverse events (AEs), Grade 4 AEs, or AEs leading to discontinuation were reported. Most AEs were Grade 1 or 2 and consistent with known safety profiles.

**Table 1.**
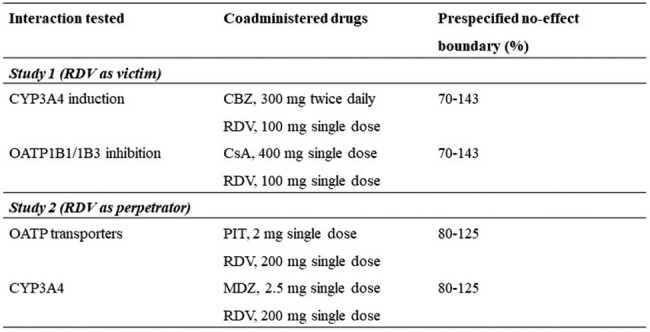
Clinical RDV DDI Study Design Characteristics RDV, remdesivir; DDI, drug-drug interaction; CYP3A4, cytochrome P450 3A4; CBZ, carbamazepine; OATP, organic anion-transporter polyprotein; CsA, cyclosporin A; PIT, pitavastatin; MDZ, midazolam.

**Conclusion:**

OATP 1B1/1B3 inhibition increased plasma PK exposures to RDV and GS-704277, but these increases remained within the range of exposures shown to be safe in Phase 3 studies and were not considered clinically relevant. CYP3A induction did not impact plasma PK exposures to RDV or metabolites. Low potential for RDV as a perpetrator of DDIs with CYP3A and OATP1B1/1B3 substrates was confirmed. For these DDIs, dose adjustments are not necessary with RDV.

**Disclosures:**

**Chi-Chi Peng, PhD**, Gilead Sciences, Inc.: Employee|Gilead Sciences, Inc.: Stocks/Bonds **Mark Shelton, PharmD**, Certara, Inc.: Employee|Gilead Sciences, Inc.: Former Employee **Anuja Raut, MS, MS**, Gilead Sciences, Inc.: Employee|Gilead Sciences, Inc.: Stocks/Bonds **Anna Kwan, BS**, Gilead Sciences, Inc.: Employee|Gilead Sciences, Inc.: Stocks/Bonds **Kanika Chhabra, MS**, Gilead Sciences, Inc.: Employee|Gilead Sciences, Inc.: Stocks/Bonds **Gong Shen, PhD**, Gilead Sciences, Inc.: Employee|Gilead Sciences, Inc.: Stocks/Bonds **Deqing Xiao, PhD**, Gilead Sciences, Inc.: Employee|Gilead Sciences, Inc.: Stocks/Bonds **Aryun Kim, PharmD**, Gilead Sciences, Inc: Company employee|Gilead Sciences, Inc: Stocks/Bonds **Sharline Madera, MD, PhD**, Gilead Sciences, Inc.: Employee|Gilead Sciences, Inc.: Stocks/Bonds **Robert H. Hyland, DPhil**, Gilead Sciences, Inc.: Employee|Gilead Sciences, Inc.: Stocks/Bonds **Helen Winter, PhD**, Gilead Sciences, Inc.: Employee|Gilead Sciences, Inc.: Stocks/Bonds **Rita Humeniuk, PhD**, Gilead Sciences, Inc.: Employee|Gilead Sciences, Inc.: Stocks/Bonds

